# Tim-3 inhibits macrophage control of Listeria monocytogenes by inhibiting Nrf2

**DOI:** 10.1038/srep42095

**Published:** 2017-02-16

**Authors:** Zhiding Wang, Dejun Sun, Guojiang Chen, Ge Li, Shuaijie Dou, Renxi Wang, He Xiao, Chunmei Hou, Yan Li, Jiannan Feng, Beifen Shen, Gencheng Han

**Affiliations:** 1 Department of Biomedicine, Institute for Regeneration Medicine, Jilin University, Changchun 130021, China; 2 Department of Immunology, Beijing Institute of Basic Medical Sciences, Beijing 100850, China

## Abstract

T cell immunoglobulin mucin-3 (Tim-3) is an immune checkpoint inhibitor and its dysregulation has been related to T cell tolerance and many immune disorders, such as tumors and infection tolerance. However, the physiopathology roles of Tim-3 in innate immunity remain elusive. Here, we demonstrate that Tim-3 inhibits macrophage phagocytosis of *L. monocytogenes* by inhibiting the nuclear erythroid 2-related factor 2 (Nrf2) signaling pathway and increases bacterial burden. Tim-3 signaling promotes Nrf2 degradation by increasing its ubiquitination and, as a result, decreasing its nuclear translocation. CD36 and heme oxygenase-1 (HO-1), two downstream molecules in the Tim-3-Nrf2 signaling axis, are involved in the Tim-3- mediated immune evasion of *L. monocytogenes* both *in vitro* and *in vivo*. We here identified new mechanisms by which Tim-3 induces infection tolerance. By modulating the Tim-3 pathway, we demonstrate the feasibility of manipulating macrophage function as a potent tool for treating infectious diseases, such as Listeria infection.

Tim-3 was first identified on differentiated Th1 cells and shown to induce T cell apoptosis by binding to its ligand galectin-9^1^. Later, it was found that Tim-3 is also expressed on activated Th17 and Tc1 cells^2^. As dysregulated overexpression of Tim-3 is related to immune disorders, such as tumors and infections tolerance, Tim-3 is now considered as a therapeutic target^3^. Recently, Tim-3 was shown to also act as an immune checkpoint regulator for innate immune cells^4^. For example, Chiba *et al*.^5^ reported that tumor-infiltrating dendritic cells show increased Tim-3 expression and suppress nucleic acid-mediated innate immune responses. We previously found that Tim-3 inhibits LPS-induced NF-kB activation in macrophages^6^ and shapes macrophage polarization^7^. Innate immune cells play critical roles both in the first line of immune defense against infection and in the activation of the adaptive immune response. Investigations of the underlying mechanisms by which Tim-3 regulates innate immune responses are of great interest.

*Listeria monocytogenes*, a Gram-positive bacterium, infects a wide range of cell types, such as macrophages and epithelial cells, and causes listeriosis in humans. It can cross the intestinal, placental, and blood-brain barriers, leading, respectively, to gastroenteritis, maternofetal infections, and meningoencephalitis^8^. Early resistance to infection is attributed to the production of interferon-γ by natural killer cells and the resultant activation of macrophages^9^. Macrophages are generally thought to be the principal cells responsible for the killing of *L. monocytogenes*. Unfortunately, a key feature of the virulence of *L. monocytogenes* is its ability to evade the killing mechanisms of professional and non-professional phagocytic host cells, especially macrophages. It is therefore of great value to investigate the mechanisms by which *L. monocytogenes* escapes from macrophage-mediated immune defense^10^.

During infection, Tim-3 expression has been reported to be upregulated on macrophages/monocytes and this inhibits their activation^11^,^12^. The physiopathological role of Tim-3 on T cells has been widely investigated^13^. However, little is known about whether and, if so, how Tim-3 induces macrophage tolerance during infection. Macrophage responses are associated with the production of reactive oxygen species (ROS) during infection^14^, which is coupled to a countervailing oxidative stress response regulated by Nrf2^15^. Nrf2 is a basic leucine zipper protein and, under unstressed conditions, is constitutively ubiquitinated and rapidly degraded by the proteasome^16^. Oxidative stress and other stimuli disrupt Keap1-Cul3-mediated ubiquitination. Then deubiquitinated Nrf2 accumulates in the cytoplasm and travels to the nucleus, where it binds to antioxidant response elements and initiates transcription of genes coding for antioxidative proteins^17^, such as CD36, a scavenger receptor involved in pathogen phagocytosis^18^, and the cytoprotective enzyme heme oxygenase-1 (HO-1), which confers host cell homeostasis and protection against a variety of pathologic conditions, including endotoxic shock and bacterial sepsis^19^. Due to the critical role of Nrf2 in immune-driven mechanisms, determining how its expression and function are regulated will provide much needed insights into infection tolerance.

Here, we demonstrate that Tim-3 functions as an immune checkpoint inhibitor of macrophages and dampens macrophage-mediated phagocytic and clearance of *L. monocytogenes* by inhibiting the Nrf2-CD36/HO-1 signaling pathway. We here provide new insights into the role of Tim-3 in disease tolerance and its possible therapeutic use.

## Materials and Methods

### Mice

Male C57BL/6 mice (6- to 8-weeks-old) were obtained from Jackson Laboratory (Bar Harbor, ME, USA). Tim-3 transgenic mice were generated in the Transgenic Core Facility of Cyagen Biosciences Inc., Guangzhou, China by overexpressing Tim-3 under the control of the CMV promoter; incorporation was confirmed by PCR and Tim-3 expression on macrophages and other cells was confirmed using flow cytometry^20^. All mice were bred and maintained in our facilities under specific pathogen-free conditions and were treated in strict compliance with the guidelines for the care and use of laboratory animals set out by the Beijing Institute of Basic Medical Sciences and the protocol was approved by the Committee on the Ethics of Animal Experiments of the Beijing Institute of Basic Medical Sciences (Permit number: AMMS2015-0165). All efforts were made to minimize suffering.

### Cell culture

The mouse macrophage cell line RAW264.7 and the human embryonic kidney cell line HEK-293-T were obtained from the American Type Culture Collection (ATCC, Manassas, VA, USA). RAW264.7 cells stably knocked down for Tim-3 were generated in our laboratory, as described previously^6^. Mouse peritoneal macrophages were collected from peritoneal lavage fluid by flow cytometry sorting, as described previously^21^. All cells were maintained in Dulbecco’s modified Eagle’s medium supplemented with 10% heat-inactivated fetal calf serum, erythromycin (5 μg/ml), and streptomycin (50 μg/ml) (all from Gibco) in a humidified 5% CO_2_ atmosphere at 37 °C. For transient expression, the vector (pcDNA3.1) alone or plasmids coding for full length human Tim-3 (Tim3-pcDNA3.1) or a human Tim-3 construct lacking the tail region (hTim3-ΔIC-pcDNA3.1) were transfected into HEK-293-T cells (ATCC) for 48 h. For Nrf2 knockdown, the Nrf2 siRNA (Santa Cruz) were transfected into RAW264.7 cells for 48 h. For Tim-3 blockade studies, the cells were incubated for 12 h (for real-time PCR and FACS) or for 15 min (for Western blotting and co-immunoprecipitation studies) with 20 μg/ml of either “soluble Tim-3” (the extracellular domains of Tim-3 linked to His-tagged thioredoxin; sTim-3-Trx) or His-tagged Trx, both prepared as described previously^6^, then were collected for analysis. For SSO (Sulfosuccinimidyl oleate sodium, Santa Cruz) inhibition, 20 μM SSO were incubated with cells for 12 h. For Snpp-IX (Santa Cruz) inhibition, 5 μM Snpp-IX were incubated with cells for 12 h. For PR619 (Selleck) inhibition^22^, 20 μM PR619 were incubated with cells for 12 h.

### Bacterial phagocytosis assay *in vitro*

*L. monocytogenes* were kindly provided by Professor Jiyan Zhang, Beijing Institute of Basic Medical Sciences, China. To measure *L. monocytogenes* phagocytosis by RAW264.7 macrophages, the bacteria were incubated overnight at 37 °C in brain heart infusion (BHI) medium supplemented with erythromycin (5 μg/ml) and streptomycin (50 μg/ml) (all from Sigma Aldrich), then an aliquot (1 ml) was washed twice with sterile PBS and resuspended in PBS for infection. *L. monocytogenes* was stained for 15 min at 37 °C with 100 mM carboxyfluorescein diacetate succinimidyl ester (CFSE; Invitrogen), then washed with PBS. CFSE labeling did not affect bacterial viability.

For flow cytometry analysis to measure phagocytosis *in vitro*, CFSE-labeled *L. monocytogenes* (2 × 10^7^ CFU) were then added to cultured RAW264.7 cells (1 × 10^6^ cells per well in a 6-well-plate) and the cultures incubated for 2 h. The macrophages were collected and washed, then analyzed by BD FACS Caliber^23^.

For confocal laser scanning microscope (CLSM)^24^ to measure phagocytosis *in vitro*, RAW264.7 cells (1 × 10^6^ cells on 15mm Glass Bottom Cell Culture Dish) were pre-incubated with control and sTim-3 for 12 h, then CFSE-labeled *L. monocytogenes* (2 × 10^7^ CFU) were added and cultured for another 2 h. After infection, the macrophages were washed twice, and then fixed using 4% formaldehyde and counterstained with Hoechst 33342. Images were obtained by Carl Zeiss LSM 880, the objective is 40 × NA0.75.

### Bacterial killing assay *in vitro*

For bacteria killing assay^25^, *L. monocytogenes* (2 × 10^7^ CFU) were added to RAW264.7 cells (1 × 10^6^ cells on 6-well plate) and infected for 2 h. After infection, the cells were washed twice and were cultured with DMEM medium containing 100 μg/ml Ampicillin to kill extracellularly located bacteria in the presence of sTim-3 (20 μg/ml) or same amount of control protein for 24 h. After stimulation, the cells were homogenized in PBS containing 0.05% Triton X-100, then six 10-fold serial dilutions (undiluted to 10^5^ dilution) of each were prepared in sterile PBS. A pre-dried BHI plate was divided into six sections, one for each dilution, by drawing lines on the bottom, then a 10 μL sample of each dilution was added to the corresponding section on the plate. When the drops were dry, the plate was inverted and incubated at 37 °C overnight, then colonies were counted to determine numbers of bacteria.

For confocal laser scanning microscope (CLSM) to measure bacteria killing *in vitro*^26^, RAW264.7 cells were infected with CFSE labeled bacteria, then washed and incubated with control or sTim-3 protein for 24 h as above killing assay. Finally cells were fixed with 4% formaldehyde and counterstained with Hoechst 33342. Images were obtained by Carl Zeiss LSM 880, the objective is 40 × NA0.75.

### Western blotting

For Western blotting, cells were harvested and lysed in lysis buffer (50 mM Tris-HCl, pH 8.0, 150 mM NaCl, 0.5% NP-40, 10% glycerol, 0.1 mM DTT, 0.2 mM NaVO_3_) supplemented with the proteasome inhibitor MG132 (5 μM) and protease and phosphatase inhibitors (all from Sigma Aldrich). For nuclear and cytoplasmic extract isolation, the nuclear extraction was prepared using an NE-PER Nuclear Cytoplasmic Extraction Reagent kit (Pierce, Rockford, IL, USA) according to the manufacturer’s instruction. Briefly, the treated cells were washed twice with cold PBS and centrifuged at 6000 rpm for 30 s. The cell pellet was suspended in 200 μl of cytoplasmic extraction reagent I by vortexing. The suspension was incubated on ice for 10 min followed by the addition of 11 μl of a second cytoplasmic extraction reagent II, vortexed for 5 s, incubated on ice for 1 min and centrifuged for 5 min at 13000 rpm. The supernatant fraction (cytoplasmic extract) was transferred to a pre-chilled tube. The insoluble pellet fraction, which contains crude nuclei, was resuspended in 100 μl of nuclear extraction reagent by vortexing during 15 s and incubated on ice for 10 min, then centrifuged for 10 min at 13 000 rpm. The resulting supernatant, constituting the nuclear extract, was used for the subsequent experiments.

Samples were then boiled for 3 min in reducing SDS sample buffer, electrophoretically separated on a 10% sodium dodecyl sulfate polyacrylamide gel, and transferred to a polyvinylidene difluoride membrane, which was then blocked by incubation for 1 h at room temperature in 5% fat-free dry milk in Tris-buffered saline containing 0.1% Tween 20 (TBST). The blots were then incubated overnight at 4 °C with a 1:1000 dilution in TBST containing 5% BSA (TBST/BSA) of rabbit antibodies against Nrf2^27^,^28^ (Abcam, ab31163), mouse antibodies against β-tubulin (Cell Signaling Technology), or rabbit antibodies against ubiquitin (Abcam), washed for 30 min with TBST, and incubated for 1 h at room temperature with a 1:20,000 dilution in TBST/BSA of alkaline phosphatase-conjugated goat anti-rabbit IgG or anti-mouse IgG antibodies (KPL, USA).

### Immunoprecipitation

For immunoprecipitation (IP) assays, the cells were washed once with PBS, then solubilized by shaking at 4 °C for 30 min in 500 μl of cold lysis buffer supplemented with the proteasome inhibitor MG132 (5 μM) and protease and phosphatase inhibitors (all from Sigma Aldrich), then the lysate was clarified by 15 min centrifugation at 12,000 g at 4 °C. The clear cell lysate was pre-treated by incubation for 3 h at 4 °C with normal IgG and protein A/G agarose beads (Roche), followed by centrifugation, then a sample of the supernatant containing 500 μg of protein was incubated overnight at 4 °C with 0.5 μg of rabbit anti-Nrf2 antibodies (H-300, Santa Cruz), then for 3 h at 4 °C with 50 μl of protein A/G agarose beads (Roche). The beads were then washed 3 times with lysis buffer and bound protein eluted by boiling for 3 min in reducing SDS sample buffer and used for Western blotting.

### High-content screening

The human A549 cell line expressing Nrf2 linked to enhanced green fluorescent protein (Nrf2-eGFP), generated in our laboratory, was added to 96-well assay plates (black plates with clear bottom), then the cells were transfected with hTim3-pcDNA3.1 or vector (pcDNA3.1) for 48 h, after which 20 μM menadion (vitamin K3), an Nrf2 nuclear translocation stimulator, was added for 6 h, then the cells were fixed with 4% paraformaldehyde and the nucleus labeled with Hoechst 33342. The cells were then imaged and analyzed using a GE IN Cell Analyzer 2000 High-Content Cellular Analysis System (GE Healthcare Bio-Sciences Corp.). To measure the activation of Nrf2, the nucleus/cytosol eGFP signal intensity ratio was calculated.

### Semi-quantitative reverse transcription–polymerase chain reaction

Gene expression was analyzed by two-step qRT–PCR. Total RNA was extracted from mouse macrophages using TRI reagent (Sigma), following the manufacturer’s instructions RNA was reverse-transcribed in a 20 μl reaction volume (42 °C, 30 min; 95 °C, 5 min) using a QuantiTect Reverse Transcription Kit (Qiagen, Valencia, CA, USA), then cDNA was amplified using a SYBR Green I Master mix (Roche, Basel, Switzerland) and a LightCycler 480 PCR system (Roche). All tests were carried out on duplicate reaction mixtures in 96-well plates. The relative expression of the gene of interest was determined using the 2^−ΔΔCt^ method, with 18 S ribosomal mRNA (18 S) as the internal control. The primers used for mouse Nrf2, HO-1, CD36, Il-1β and 18 S were:

Nrf2: Sense 5′-TAGATGACCATGAGTCGCTTGC-3′

Anti-sense 5-GCCAAACTTGCTCCATGTCC-3′

HO-1: Sense 5′-CACGCATATACCCGCTACCT-3′

Anti-sense 5′-CCAGAGTGTTCATTCGAGCA-3′

CD36: Sense 5′-TTTCCTCTGACATTTGCAGGTCTA-3′

Anti-sense 5′-AAAGGCATTGGCTGGAAGAA-3′

IL-1β, Sense 5′-TGGGCCTCAAAGGAAAGA-3′

Anti-sense 5′-GGTGCTGATGTACCAGTT-3′

18 S: Sense 5′-TTGACGGAAGGGCACCACCAG-3′

Anti-sense 5′-GCACCACCACCACGGAATCG-3′.

### *L. monocytogenes* studies *in vivo*

For infection, each mouse was injected intraperitoneally (i.p.) with *L. monocytogenes* (5 × 10^7^ CFU) and sacrificed at day 3 post-infection to determine *L. monocytogenes* burden. To determine Listeria burden, peritoneal lavage fluid was collected and the spleen was homogenized in PBS containing 0.05% Triton X-100, then six 10-fold serial dilutions (undiluted to 10^5^ dilution) of each were prepared in sterile PBS. A pre-dried BHI plate was divided into six sections, one for each dilution, by drawing lines on the bottom, then a 10 μL sample of each dilution was added to the corresponding section on the plate. When the drops were dry, the plate was inverted and incubated at 37 °C overnight, then colonies were counted to determine numbers of bacteria. The liver was used for histological analysis. Briefly, the liver were excised and fixed in 10% buffered formalin, then 5 μm sections were prepared and stained with H&E for microscopic observations.

In some studies, *L. monocytogenes*-infected mice were injected i.p. with 200 μg of Trx or sTim-3-Trx on the day of infection, then peritoneal macrophages, spleen, and peritoneal fluid were collected 3 days later and tested.

### Statistical analysis

Data are expressed as the mean ± standard deviation. Differences between groups were analyzed using the Kruskal–Wallis test and analysis of variance (ANOVA). A P value less than 0.05 was considered significant. SPSS software (version 20.0) was used for all statistical procedures.

## Result

### Tim-3 inhibits macrophage phagocytosis of *L. monocytogenes*

**Figure 1:**
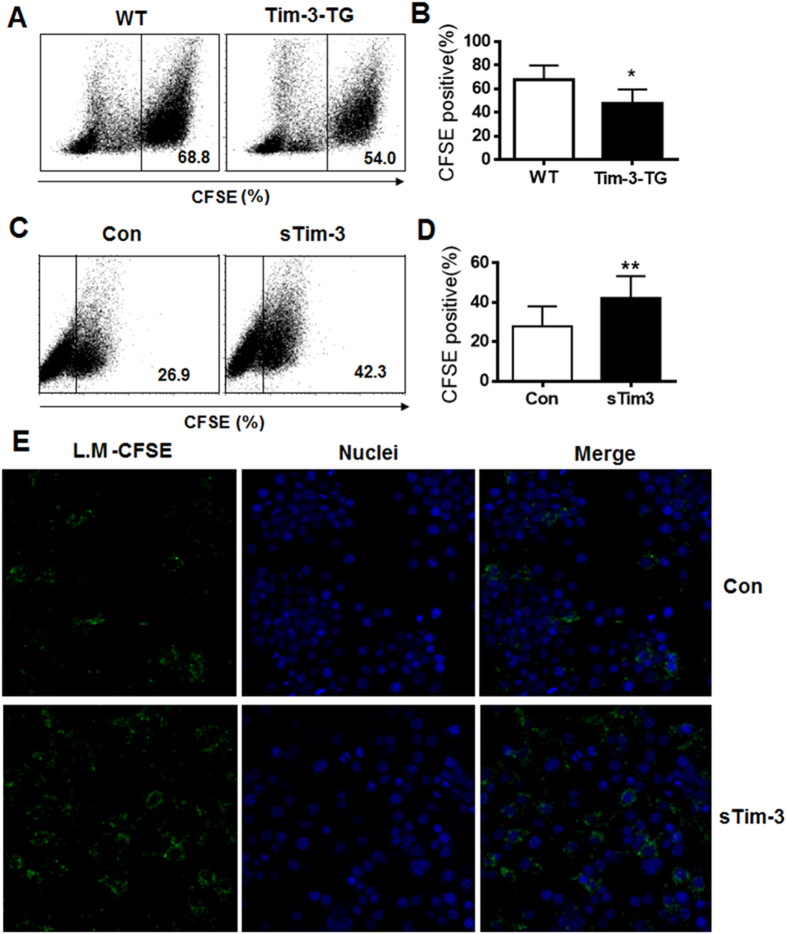
Tim-3 signaling inhibits macrophage control of *L. monocytogenes in vitro*. (**A**,**B**) *L. monocytogenes* (10^7^ CFU) were stained with CFSE for 15 min before being added to cultured peritoneal macrophages from wild type (WT) or Tim-3-TG C57BL/6 mice, then, after 2 h infection, the cells were washed and examined for CFSE staining by flow cytometry. (**A**) shows a typical example, while (**B**) shows the mean ± SD obtained with 5 macrophage samples from different mice *p < 0.05. (**C** and **D**) *L. monocytogenes* were stained with CFSE as above and added to cultured RAW264.7 cells in the presence of 20 μg/ml of sTim-3-Trx (sTim-3) or Trx control (Con) for 2 h, then the cells were collected and examined for CFSE staining by flow cytometry. (**C**) shows a typical example, while (**D**) shows the result for triplicate wells expressed as the mean ± SD; *p < 0.05.** p < 0.01. (**E**) *L. monocytogenes* were stained with CFSE and added to RAW264.7 cells in the presence of 20 μg/ml of sTim-3-Trx (sTim-3) or Trx (Con) for 2 h, then the cell were dyed by Hoechst 33342 (blue) and were imaged by confocal laser scanning microscope. Similar results were obtained in three independent experiments.

We previously found that Tim-3 inhibits macrophages activation and induces its tolerance. However, it was not known whether Tim-3 regulates the phagocytic activity of macrophages. To test this, we labeled *L. monocytogenes* with CFSE and added them to cultures of peritoneal macrophages isolated from wild type (WT) or Tim-3 transgenic (Tim-3-TG) mice or to cultured RAW264.7 cells with soluble Tim-3 protein (sTim-3) as soluble Tim-3 is widely used to block the Galectin-9/Tim-3 interaction^29^,^30^, then, after 2 h incubation, *L. monocytogenes*- CFSE- containing cells were measured by flow cytometry as described previously^21^. The results in Fig. 1A and B show that transgenic overexpression of Tim-3 inhibited macrophage phagocytosis of Listeria, while Fig. 1C and D show that blockade of Tim-3 signaling by soluble Tim-3 enhanced phagocytosis. These data show that Tim-3 signaling inhibits macrophage phagocytosis of *L. monocytogenes in vitro*. To test this in a direct way, we labeled *L. monocytogenes* with CFSE, and after co-culture with macrophages, examined macrophage phagocytosis of *L. monocytogenes* using confocal laser scanning microscope as described previously^24^. The data in Fig. 1E showed that blockade of Tim-3 signaling significantly increased macrophage phagocytosis of *L. monocytogenes* which is consistent with the result of flow cytometry analysis.

### Tim-3 inhibits macrophage phagocytosis of Listeria by a mechanism involving Nrf2

**Figure 2:**
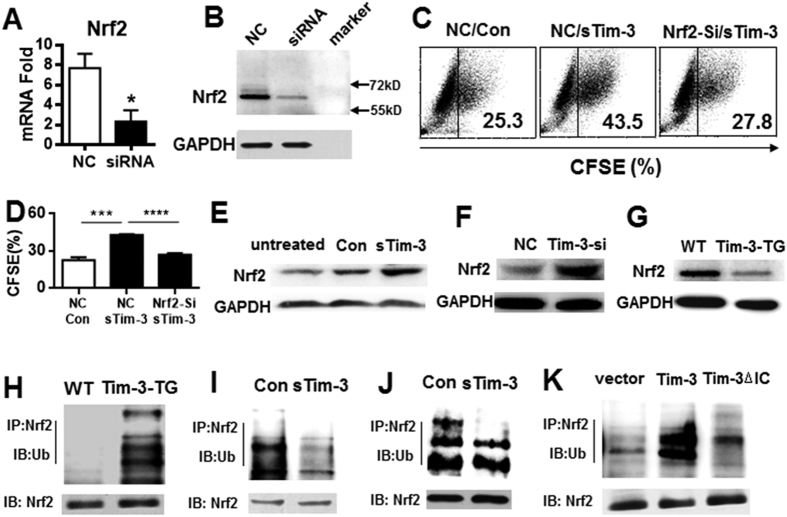
Tim-3 inhibits macrophage control of *L. monocytogenes* via Nrf2. (**A**,**B**) Nrf2 mRNA (**A**) and protein (**B**) levels in RAW264.7 cells silenced for Nrf2 expression; (**C**,**D**) RAW264.7 cells treated with nonsense siRNA (NC) or Nrf2 siRNA were infected with CFSE-labeled *L. monocytogenes* for 2 h in the presence of 20 μg/ml of soluble sTim-3-Trx (sTim-3) or Trx control (Con), then the cells were analyzed for CFSE staining by flow cytometry; (**C**) shows a typical result, while (**D**) show the mean ± SD for triplicate wells. *p < 0.05. Similar results were obtained in three independent experiments. (**E**) RAW264.7 cells were incubated with medium or 20 μg/ml of sTim-3-Trx (sTim-3) or Trx control (Con) for 15 min, then were collected and analyzed for Nrf2 expression by Western blotting. (**F**) Normal RAW264.7 cells (NC) or RAW264.7 cells silenced for Tim-3 (Tim-3-si) were lysed and analyzed for Nrf2 expression by Western blotting. (**G**) Peritoneal macrophages isolated from WT or Tim-3-TG mice were analyzed for Nrf2 expression by Western blotting. (**H**) Peritoneal macrophages isolated from WT or Tim-3-TG mice were lysed, then Nrf2 protein was precipitated with specific antibody and examined for ubiquitination by Western blotting. (**I**) RAW264.7 cells were incubated with 20 μg/ml of sTim-3-Trx (sTim-3) or Trx control (Con) for 15 min, then were lysed and Nrf2 protein precipitated with specific antibody and examined for ubiquitination by Western blotting. (**J**) *L monocytogenes*- infected mice were injected intraperitoneally with 200 μg of sTim-3-Trx (sTim-3) or Trx control (Con) on the day of infection, then peritoneal macrophages were collected 3 days later and lysed, then Nrf2 protein was precipitated with specific antibody and examined for ubiquitination by Western blotting. (**K**) HEK-293-T cells transfected with a plasmid coding for Tim-3 or Tim-3 lacking the intracellular tail (Tim-3ΔIC) or the vector control were lysed and Nrf2 protein precipitated with specific antibody and examined for ubiquitination by Western blotting. In (**E**–**K**), the results shown are representative of those obtained in three independent experiments.

We next investigated the mechanism by which Tim-3 inhibits macrophage phagocytosis of Listeria and found that Nrf2, a transcription regulator of anti-oxidant genes, is involved in Tim-3 inhibition of macrophage phagocytosis. Nrf2 knockdown (Fig. 2A and B) blocked the soluble Tim-3 -induced increased phagocytosis of *L. monocytogenes* (Fig. 2C and D). Furthermore, both blockade of Tim-3 signaling using soluble Tim-3 (Fig. 2E) and silencing of Tim-3 (Fig. 2F) in RAW264.7 cells all led to increased Nrf2 protein expression, while transgenic overexpression of Tim-3 in mice resulted in decreased Nrf2 expression in macrophages (Fig. 2G). These data show that Nrf2 is involved in Tim-3-mediated inhibition of macrophage phagocytosis of *L. monocytogenes* and that Tim-3 expression or activity inhibits Nrf2 expression at the protein level. As we did not find any inhibition by Tim-3 on Nrf2 mRNA levels (data not shown), we speculated that Tim-3 inhibits Nrf2 expression by post-transcriptional mechanisms, as Nrf2 activity is regulated by ubiquitination^17^. To test this, we precipitated Nrf2 protein from peritoneal macrophages from WT mice or Tim-3-TG mice (Fig. 2H), from RAW264.7 cells with or without blockade using soluble Tim-3 (Fig. 2I), and from peritoneal macrophages taken from *L. monocytogenes* infected mice with or without Tim-3 blockade (Fig. 2J). Our results showed that Tim-3 signaling significantly increases Nrf2 ubiquitination in macrophages (Fig. 2H), while blockade of Tim-3 signaling, both in macrophages *in vitro* (Fig. 2I) and in *L. monocytogenes-*infected mice *in vivo* (Fig. 2J), decreased Nrf2 ubiquitination. As ubiquitination of Nrf2 inhibits its activity^17^, our data suggest that Tim-3 may inhibit Nrf2 by promoting its ubiquitination. Finally, to demonstrate that it was indeed Tim-3 signaling that mediated the suppression of Nrf2 activity, we generated a Tim-3 construct lacking the intracellular region (Tim-3-ΔIC) and transfected plasmids coding for Tim-3 or Tim-3-ΔIC into HEK-293-T cells, then examined Nrf2 ubiquitination. As shown in Fig. 2K, deletion of the tail significantly decreased Nrf2 ubiquitination. Together, these data show that Nrf2 plays a critical role in the Tim-3-mediated inhibition of macrophage phagocytosis of *L. monocytogenes* and suggest that Tim-3 may decrease Nrf2 protein level by inducing Nrf2 ubiquitination and degradation by the proteasome.

### Tim-3 decreases nuclear translocation of Nrf2

**Figure 3:**
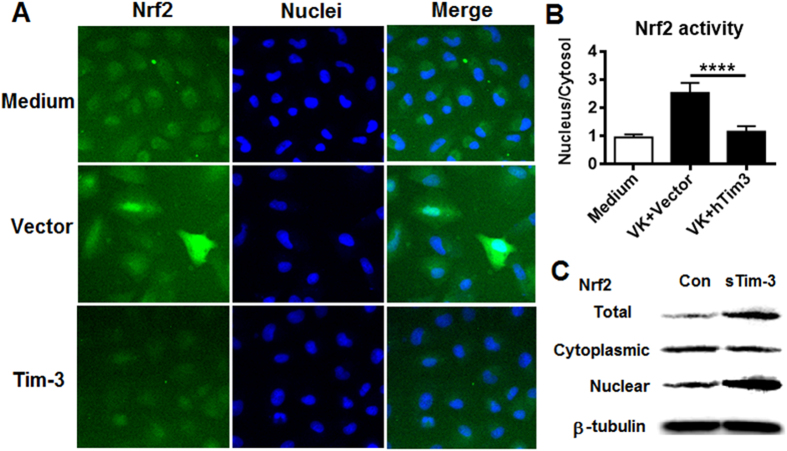
Tim-3 inhibits Nrf2 nuclear translocation. A549 cells expressing GFP-labeled Nrf2 were transfected with hTim3-pcDNA3.1 or vector control for 48 h, then were incubated with 20 μM menadione (VK) for 30 min and imaged and analyzed on a GE IN Cell Analyzer 2000. The activation of Nrf2 was expressed as the nucleus/cytosol GFP signal ratio. (**A**) Representative images showing VK-induced nuclear translocation of Nrf2. (**B**) Summarized data for the PCTACT in the presence of VK. The results, which are representative of those obtained in three independent assays, are the mean ± SD for triplicate wells; ***p < 0.001. (**C**) RAW264.7 cells were incubated with 20 μg/ml of sTim-3-Trx (sTim-3) or Trx control (Con) for 15 min, then nuclear and cytoplasmic extract were isolated to analyze for Nrf2 protein level by Western blotting.

To further examine the effects of Tim-3 on Nrf2 activity, A549 cells transfected with the Nrf2-EGFP vector were used; in these cells, Nrf2 is translocated into the nucleus in response to stimulation with menadion (vitamin K3)^31^. When these cells were transfected with vector or hTim3-pcDNA3.1 for 48 h, then incubated with menadion for 30 min, Nrf2 nuclear translocation was considerably lower in the Tim-3-transfected cells than that in the vector-transfected cells (Fig. 3A and B). Here we also examined the effects of Tim-3 on Nrf2 nuclear translocation by nuclear and cytoplasmic extract and isolation assay. The data in Fig. 3C showed that the Tim-3 blockade increased Nrf2 mainly appears in the nuclear, suggesting that Tim-3 signaling decreases the nuclear translocation of Nrf2. Although detail mechanisms remain to be determined, these data demonstrate the suppressive effect of Tim-3 on the nuclear translocation of Nrf2.

### CD36 and HO-1, two molecules with Nrf2-regulated expression, are involved in Tim-3-mediated inhibition of macrophage phagocytosis of *L. monocytogenes*

**Figure 4:**
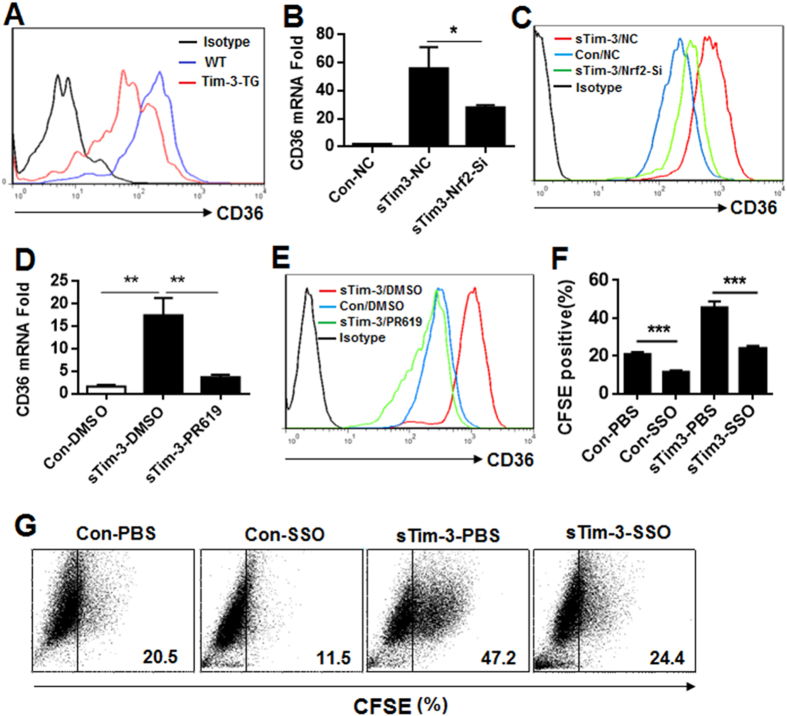
Tim-3 inhibits macrophage control of *L. monocytogenes* via the Nrf2-CD36 signaling axis. (**A**) Peritoneal macrophages isolated from WT or Tim-3-TG mice were examined for CD36 expression by flow cytometry. (**B**,**C**) RAW264.7 cells (NC) or Nrf2-knockdown (Nrf2-si) RAW264.7 cells were incubated with 20 μg/ml of soluble sTim-3-Trx (sTim-3) or Trx control (Con) for 12 h (for PCR) or for 24 h (for FACS), then were collected and analyzed for CD36 mRNA expression by real-time PCR (**B**) and for CD36 protein expression (**C**) by flow cytometry. (**D**,**E**) RAW 264.7 cells were incubated with 20 μg/ml of sTim-3-Trx (sTim-3) or Trx control (Con) and either DMSO (vehicle) or the deubiquitination inhibitor PR619 (20 μM) for 12 h (for PCR) or for 24 h (for FACS), then were collected and analyzed for CD36 mRNA expression by real-time PCR (**D**) and for CD36 protein expression (**E**) by flow cytometry. (**F**,**G**) RAW264.7 cells infected with CFSE-labeled *L. monocytogenes* were incubated with PBS or the CD36 inhibitor SSO (20 μM) for 12 h in the presence of 20 μg/ml of sTim-3-Trx (sTim-3) or Trx control (Con), then were collected and analyzed for CFSE staining in macrophages by flow cytometry. (**G**) Representative data showing CFSE staining of the macrophages in (**F**). In (**B**,**D**,**F**), the results are expressed as the mean ± SD of triplicate wells. *p < 0.05; **p < 0.01; ***p < 0.001. The results shown are representative of those obtained in three independent experiments.

CD36 is a scavenger receptor involved in pathogen phagocytosis^18^. As shown in Fig. 4A, examination of peritoneal macrophages from WT and Tim-3-TG mice showed that Tim-3 overexpression led to decreased CD36 expression. As CD36 expression is regulated by Nrf2^32^, it was reasonable to hypothesize that Tim-3 inhibits macrophage control of *L. monocytogenes* by inhibiting the Nrf2-CD36 pathway. To test this, we used RAW264.7 cells with or without silencing of Nrf2, as in Fig. 2A and B, and found that CD36 mRNA levels (Fig. 4B) and protein level (Fig. 4C) in RAW264.7 cells were increased following incubation for 12 h with sTim3 and that this effect was inhibited by Nrf2 silencing (Fig. 4B and C). Furthermore, the data in Fig. 4D and E showed that the increase in CD36 mRNA (Fig. 4D) and protein (Fig. 4E) levels induced by incubation of control RAW264.7 cells with soluble Tim-3 was blocked by a deubiquitination inhibitor, PR619, suggesting that Tim-3 signaling may inhibit CD36 expression by alerting the ubiquitination of Nrf2. Finally, to examine whether Tim-3 inhibited macrophage control of *L. monocytogenes* through CD36, SSO, a specific CD36 inhibitor^33^, or vehicle (PBS) was included in cultures of *L. monocytogenes*-infected RAW264.7 cells incubated for 12 h in the presence or absence of soluble Tim-3, then phagocytosis of CSFE-labeled *L. monocytogenes* was measured. As shown in Fig. 4F and G, SSO inhibited both basal and soluble Tim-3- enhanced RAW264.7 cell phagocytosis of *L. monocytogenes*. These data show that Tim-3 inhibits macrophage control of *L. monocytogenes* through the Nrf2-CD36 pathway.

**Figure 5:**
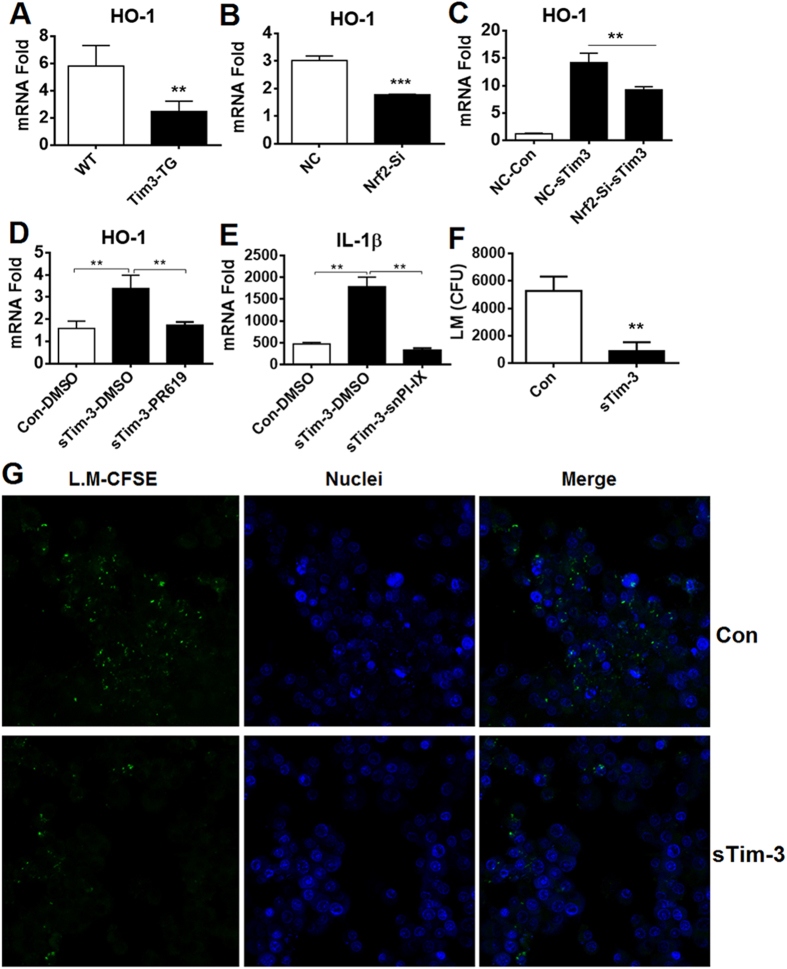
Tim-3 inhibits the HO-1-IL-1β signaling pathway in macrophages via Nrf2. (**A**) Peritoneal macrophages isolated from WT and Tim-3-TG mice were analyzed for HO-1 mRNA levels by real-time-PCR. (**B**) Control or RAW264.7 cells silenced for Nrf2 as in Fig. 2A were examined for HO-1 mRNA levels by real-time PCR. (**C**) RAW264.7 cells or Nrf2 knockdown RAW264.7 cells were incubated with 20 μg/ml of sTim-3-Trx (sTim-3) or Trx control (Con) for 12 h, then were collected and HO-1 mRNA levels examined by real-time PCR. (**D**) RAW264.7 cells were incubated with 20 μg/ml of sTim-3-Trx (sTim-3) or Trx control (Con) in the presence of PR619 (20 μM) or vehicle (DMSO) for 12 h, then were collected and HO-1 mRNA levels examined by real-time PCR. (**E**) RAW264.7 cells were incubated with 20 μg/ml of sTim-3-Trx (sTim-3) or Trx control (Con) in the presence or absence of an HO-1 inhibitor (Snpp-IX, 5 μM) or vehicle (DMSO) for 12 h, then were infected with *L. monocytogenes* (1 × 10^6^ CFU) for 12 h and IL-1β mRNA levels examined by real-time PCR. (**F**,**G**) Tim-3 blockade promotes macrophages killing of *L. monocytogenes*. Raw264.7 cells were infected with *L. monocytogenes* (**F**) or CFSE-labeled *L. monocytogenes* (**G**) for 2 h, then washed, and incubated with Ampicillin to kill extracellularly located bacteria in the presence of sTim-3 or control protein for 24 h. Macrophages were either homogenized for CFU counting (**F**) or were imaged using confocal laser scanning microscope to examine the living bacteria (CFSE-bright spot) (**G**). The data are expressed as the mean ± SD for triplicate wells; **p < 0.01; ***p < 0.001, and are representative of the results obtained in three independent experiments.

HO-1 is another molecule downstream of Nrf2 which plays an important role in bacterial phagocytosis and clearance^34^. As shown in Fig. 5A, we found that transgenic overexpression of Tim-3 in mice inhibited HO-1 expression at the mRNA level in peritoneal macrophages compared to in WT mice, while Fig. 5B shows that silencing of Nrf2 in RAW264.7 cells led to increased HO-1 mRNA levels. Furthermore, soluble Tim-3- induced upregulation of HO-1 mRNA levels in control RAW264.7 cells was inhibited by Nrf2 knockdown (Fig. 5C) or by addition of the deubiquitination inhibitor PR619 (Fig. 5D). These data show that Tim-3 signaling inhibits HO-1 expression via Nrf2. Since IL-1β is an important cytokine for protection against bacterial infection, including *L. monocytogenes* clearance *in vivo*^34^, and since its expression can be regulated by HO-1, we examined whether soluble Tim-3 had any effect on IL-1β mRNA levels in RAW264.7 cells during *L. monocytogenes* infection and found that it caused an increase that was prevented by the HO-1 inhibitor Snpp-IX^35^ (Fig. 5E). Finally, macrophages mediated bacteria killing assays were carried out *in vitro* according to the published methods^25^,^26^. Following 2 h of invasion and clearance of extracellularly located L.monocytogenes, RAW264.7 cells were stimulated with sTim-3 or control protein for 24 h. Then bacteria killing by macrophages was examined by counting the CFU (Fig. 5F) and by imaging CFSE-bright spot (green-bacterial) using confocal laser scanning microscope (Fig. 5G). Our data showed that blockade of Tim-3 significantly decreased CFU (Fig. 5F) and decreased the number of living bacteria (green spots) within infected macrophages.

### Tim-3 controls *L. monocytogenes* burden *in vivo*

**Figure 6:**
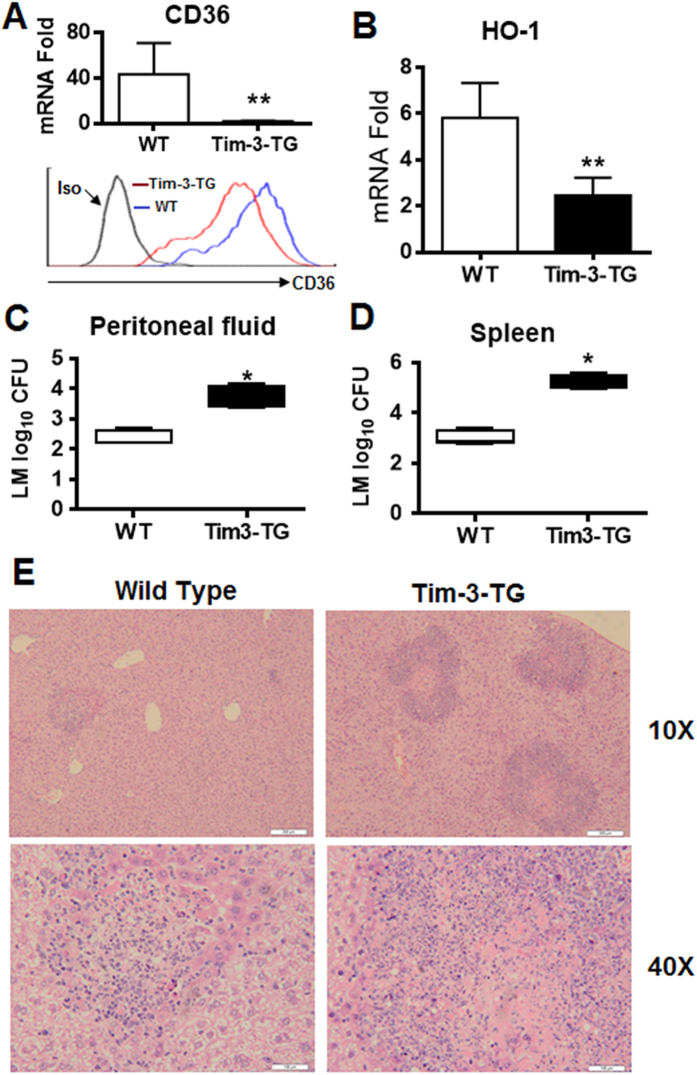
Tim-3 inhibits CD36 and HO-1 expression by macrophages *in vivo* and exacerbates *L. monocytogenes* infection. Wild type or Tim-3 transgenic mice were infected with *L. monocytogenes* (5 × 10^7^ CFU) for 3 days, then peritoneal macrophages were isolated and examined for CD36 mRNA (A up panel) by real-time PCR and protein (A lower panel) by flow cytometry and HO-1 (**B**) mRNA by real-time PCR and the bacterial burden was examined in the peritoneal fluid (**C**) and spleen homogenate (**D**). (**E**) H&E staining of liver sections, which showed much more numerous, large foci of infection in the livers of Tim-3 -TG mice compare to those scattered, small foci of infection often surrounding a core of dead hepatocytes in the livers of WT mice. In (**A**–**D**), the data shown are the mean ± SD for a group of 6 mice and are representative of results for two independent experiments using 4 or 6 mice in each group. *p < 0.05; **p < 0.01.

The above data demonstrated that Tim-3 inhibits macrophage control of *L. monocytogenes* through the Nrf2-CD36/HO-1 signaling pathways *in vitro*. To investigate whether this signaling pathway affected the progression of *L. monocytogenes* infection *in vivo*, we injected *L. monocytogenes* into WT or Tim-3-TG mice, then, after 3 days, measured CD36 and HO-1 expression in peritoneal macrophages and *L monocytogenes* burden in the spleen and peritoneal fluid. The results showed that, during *L. monocytogenes* infection, transgenic overexpression of Tim-3 led to a significant decrease in mRNA and protein level for CD36 (Fig. 6A) and mRNA level for HO-1 (Fig. 6B) in peritoneal macrophages and to a significant increase in both bacterial burden in the peritoneal fluid (Fig. 6C) and spleen (Fig. 6D) and the inflammatory response in the liver (Fig. 6E). These data show that Tim-3 signaling leads to *L. monocytogenes* infection tolerance in mice *in vivo*, and suggest that Tim-3 may induce infection tolerance by inhibiting the CD36 and HO-1 signaling pathways in macrophages.

**Figure 7:**
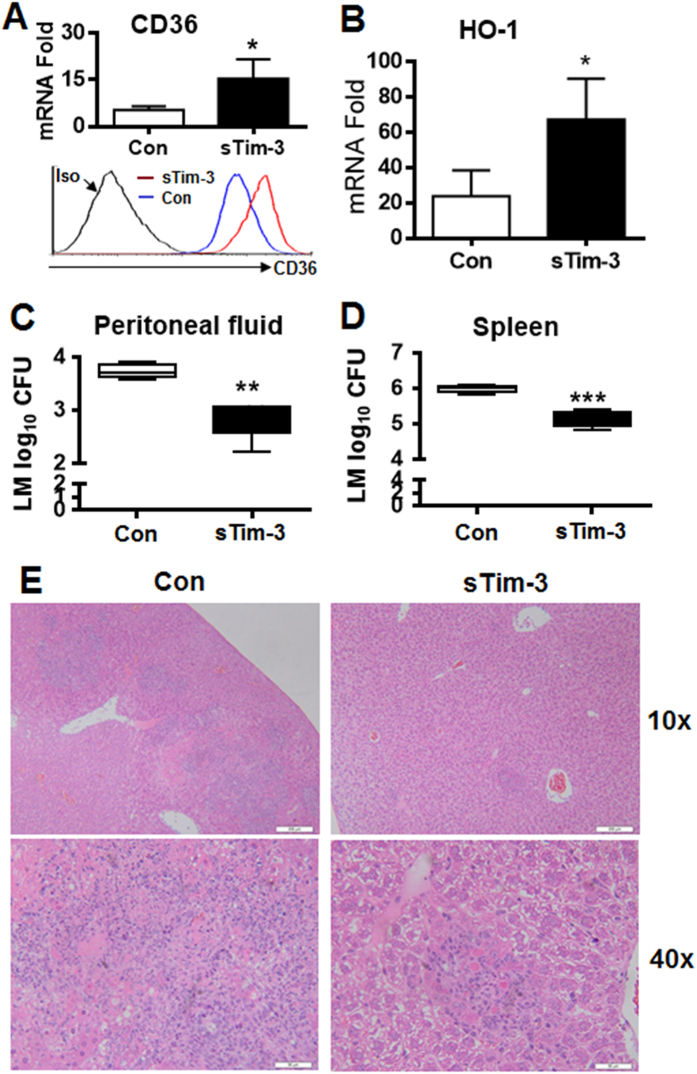
Blockade of Tim-3 *in vivo* increases CD36 and HO-1 expression by macrophages and attenuates *L. monocytogenes* infection. C57BL/6 mice were injected i.p. with *L. monocytogenes* (5 × 10^7^ CFU/mouse), then with 200 μg of soluble sTim-3-Trx (sTim-3) or Trx control (Con). Three days later, peritoneal macrophages were isolated and examined for the expression of CD36 mRNA (A up panel) by real-time PCR and protein (A lower panel) by flow cytometry and HO-1 (**B**) mRNA levels by real-time PCR and the bacterial burden was examined in the peritoneal fluid (**C**) and spleen homogenate (**D**). (**E**) H&E staining of liver sections, which showed scattered, small foci of infection often surrounding a core of dead hepatocytes in the livers of sTim-3-Trx (sTim-3) treated mice compare to those much more numerous, large foci of infection in the livers of Trx control (Con) treated control mice. In (**A**–**D**), the data shown are the mean ± SD for a group of 6 mice and are representative of results for two independent experiments using 4 or 6 mice in each group. *p < 0.05; **p < 0.01; ***p < 0.001.

To examine the role of Tim-3 signaling *in vivo* using another approach, we injected *L. monocytogenes* into WT C57BL/6 mice together with 200 μg of soluble Tim-3 or Trx control, then collected peritoneal macrophages, spleen, and peritoneal fluid 3 days later and carried out the same examinations as in Fig. 6. The results showed that i.p. injection of soluble Tim-3 led to a significant increase in mRNA and protein level for CD36 (Fig. 7A) and mRNA level for HO-1 (Fig. 7B) in peritoneal macrophages, a decreased bacterial burden in the peritoneal fluid (Fig. 7C) and spleen (Fig. 7D), and an attenuated *L. monocytogenes*-induced inflammatory response in the liver (Fig. 7E). These data further demonstrate that Tim-3 signaling inhibits the anti-*L. monocytogenes* immune response *in vivo* and suggest that Tim-3-CD36/HO-1 signaling in macrophages plays an important role in Tim-3-induced infection tolerance in mice *in vivo*. By demonstrating the effectiveness of manipulating the Tim-3 pathway *in vivo*, our data provide a new strategy for treating bacterial infectious diseases.

## Discussion

In this study, we identified new mechanisms by which Tim-3 inhibits the host immune response against *L. monocytogenes* and found that Tim-3 inhibited macrophage- mediated phagocytosis and clearance of *L. monocytogenes*. In the *in vitro* experiments, we demonstrated that Tim-3 signaling inhibits macrophage phagocytosis of L. monocytogenes by promoting Nrf2 degradation. We also found that CD36 and HO-1, two molecules with Nrf2-regulated expression, were involved in the Tim-3-induced inhibition of macrophage control of *L. monocytogenes*. In the *in vivo* experiments, blocking of the Tim-3 pathway resulted in increased CD36 and HO-1 expression in macrophages and decreased *L. monocytogenes* infection, thus identifying a new mechanism by which Tim-3 induces infection tolerance.

During infection, such as HIV or HBV infection, increased Tim-3 expression on T cells has been linked to T cell dysfunction, as demonstrated by decreased protein levels of IFN-gamma and/or Granzyme B^36^,^37^, which leads to infection tolerance^38^. However, a recent report by Gorman *et al*. showed that Tim-3 directly enhances CD8 T cell responses to acute Listeria monocytogenes infection^39^. Although this study showed that the roles of Tim-3 in regulating T cell responses is more complex, it does not invalidate all the previous work showing that Tim-3 functions as an inhibitory receptor^40^. Here we focus the roles of Tim-3 in innate immunity against *L. monocytogenes* infection. As macrophages play critical roles in bacterial recognition and defense, we examined whether and, if so, how Tim-3 is involved in macrophage-mediated bacterial phagocytosis and clearance. Interestingly, we found that blockade of Tim-3 signaling significantly increased macrophage phagocytosis of *L. monocytogenes* and demonstrated that Tim-3 inhibits macrophage control of *L. monocytogenes* by inhibiting the Nrf2-CD36/HO-1 signaling pathways. To the best of our knowledge, this is the first report showing that Tim-3 induces infection tolerance by inhibiting macrophage function.

Recognition of *L. monocytogenes* by macrophages triggers an innate immune response. Following phagocytosis of *L. monocytogenes*, macrophages can directly kill the bacteria using the ROS system. However, excessive production of ROS damage the immune cells mediated bacterial clearance^41^. Nrf2, a basic leucine zipper protein, regulates the expression of antioxidant proteins, such as CD36 and HO-1, which protect against oxidative damage and enhance bacterial phagocytosis and clearance^18^,^19^. Here we demonstrated that Tim-3 signaling inhibits the activation of Nrf2 in macrophage. A report by Harvey CJ *et al*.^42^ showed that activation of Nrf2 signaling improves bacterial clearance by alveolar macrophages. These data are consistent with our findings and show that Nrf2 plays a critical role in macrophage mediated immune defense against infection. We thus identified a new mechanism by which Tim-3 induces infection tolerance *in vivo*. As for the molecular mechanism, we found that Tim-3 signaling promoted ubiquitination of Nrf2 and inhibited its cytoplasmic accumulation and nuclear translocation. We did not investigate the detailed mechanism by which Tim-3 promotes Nrf2 ubiquitination, but showed that the C-terminal tail of Tim-3 played an important role in Tim-3-mediated Nrf2 ubiquitination and that Tim-3 inhibited CD36 and HO-1 expression in an Nrf2-dependent manner.

Nrf2 is a transcription factor for CD36, which belongs to the class B scavenger receptor family and plays a physiological role in the recognition and clearance of apoptotic cells by professional phagocytes^43^,^44^. Pathogen sensing by the cells of the innate immune system and the phagocytic clearance of bacteria is a complex process in which CD36 plays an important role. For example, CD36 contributes to macrophage-mediated clearance of *Plasmodium falciparum*-parasitized erythrocytes^45^. CD36 also acts as a co-receptor with the Toll-like receptor (TLR) 2/6 complex, which binds diacylglycerides, such as lipoteichoic acids^18^. CD36 deficiency is associated with a dysregulated cytokine response and increased mortality in experimental animal models of severe malaria^46^. In this study, we demonstrated that CD36 expression could be regulated by Tim-3 in a Nrf2-dependent manner and that Tim-3 inhibited macrophage phagocytosis of *L. monocytogenes* in a CD36-dependent manner. Our data therefore provided a new Tim-3 signaling pathway which partially explained how Tim-3 signaling regulates macrophage control of bacteria, such as *L. monocytogenes*.

HO-1 is another gene regulated by Nrf2 and plays key roles in cytoprotection, antioxidation, and anti-inflammatory responses. HO-1 deficiency causes an increased pro-inflammatory state and susceptibility to oxidative stress in humans^47^. Tachibana *et al*.^8^ demonstrated that Listeria and Brucella infections are associated with the death of trophoblast giant cells and that reduction of HO-1 expression by bacterial infection increases infectious abortions *in vivo* and cell death *in vitro*. Wegiel *et al*.^34^ found that HO-1 deficiency results in inadequate pathogen clearance, exaggerated tissue damage, and increased mortality. In this study, we found that Tim-3 inhibited HO-1 expression in macrophages via Nrf2, suggesting that Tim-3 may inhibit macrophage control of *L. monocytogenes* through HO-1. Since increased HO-1 levels enhance bacteria clearance by macrophages through pathways involving CO, NALP3, and IL-1β^34^, we examined whether Tim-3 inhibited macrophage control of *L. monocytogenes* by inhibiting IL-1β expression and found that, during *L. monocytogenes* infection, the increase in IL-1β mRNA levels caused by Tim-3 blockade using soluble Tim-3 was prevented by Snpp-IX, a HO-1 inhibitor. Indeed, in an *in vitro* bacterial killing assay, we found that blockade of Tim-3 significantly enhanced the clearance of *L. monocytogenes* by macrophages. These data suggest that, during bacterial infection, Tim-3 may inhibit IL-1β production by macrophages through HO-1 and thus attenuate macrophage-mediated bacterial clearance.

Finally, we investigated whether Tim-3 signaling inhibited macrophage control of *L. monocytogenes in vivo. L. monocytogenes* injection into WT and Tim-3-TG mice demonstrated that the Tim-3-TG mice showed decreased CD36 and HO-1 expression by peritoneal macrophages, an increased bacterial burden in the peritoneal fluid and spleen cells, and increased inflammation in the liver. In contrast, blockade of Tim-3 signaling by injection of soluble Tim-3 significantly enhanced CD36 and HO-1 expression by peritoneal macrophages, decreased the bacterial burden in the peritoneal fluid and spleen cells, and decreased inflammation in the liver. These data show that Tim-3 signaling also inhibits macrophage activity *in vivo* and may inhibit *L. monocytogenes* phagocytosis and clearance.

Roles of Tim-3 in T cells anergy or exhaustion during infection had been reported by many groups^1^,^2^,^6^. Here we demonstrated that Tim-3 may also induce infection tolerance by suppressing the activity of macrophage. As we investigated the progress of *L. monocytogenes* infection in mice within three days, we argue that, during this stage, Tim-3 signaling in innate immune cells plays a critical role in Tim-3 induced infection tolerance. Although we cannot exclude the involvement of other cells in Tim-3 attenuated bacteria clearance, our data did demonstrate that Tim-3 induced macrophage tolerance during *L. monocytogenes* infection *in vivo*.

In addition to Tim-3, other immune checkpoint inhibitors such as CTLA-4 and PD-1 are also involved in infection tolerance. For example, report showed that higher PD-1 expression is associated with unfavorable outcome in patients with sepsis^48^. Chang KC *et al*. reported that blockade of the negative co-stimulatory molecules PD-1 and CTLA-4 improves survival in primary and secondary fungal sepsis^49^. Here we demonstrated that Tim-3 blocking enhances macrophages mediated bacteria phagocytosis and clearance, suggesting that Tim-3 may have similar effects as PD-1 and CTLA-4 on immunosuppression. Therapeutic strategy targeting PD-1 and CTLA-4 are showing promising clinical benefits for immune disorders^50^,^51^. Here we provided new evidence that Tim-3 can be used as therapeutic target for infection diseases; especially for patients resistant to treatment targeting other check point inhibitors.

In summary, we have identified a new mechanism by which Tim-3 suppresses anti-*L. monocytogenes* responses. By suppressing the Nrf2-HO-1/CD36 signaling pathways in macrophages, Tim-3 inhibits macrophage control of *L. monocytogenes*. To the best of our knowledge, this is the first report showing that Tim-3 induces infection tolerance by inhibiting macrophage function. And we provide a new strategy for treating infection diseases, such as *L. monocytogenes* infection.

## Additional Information

**How to cite this article:** Wang, Z. *et al*. Tim-3 inhibits macrophage control of Listeria monocytogenes by inhibiting Nrf2. *Sci. Rep.* **7**, 42095; doi: 10.1038/srep42095 (2017).

**Publisher's note:** Springer Nature remains neutral with regard to jurisdictional claims in published maps and institutional affiliations.
